# Political Priorities, Voting, and Political Action Committee Engagement of Emergency Medicine Trainees: A National Survey

**DOI:** 10.5811/westjem.59351

**Published:** 2023-05-05

**Authors:** Rachel E. Solnick, Zachary J. Jarou, Cheryl K Zogg, Dowin Boatright

**Affiliations:** * Icahn School of Medicine at Mount Sinai, Department of Emergency Medicine, New York, New York (Current); † Michigan State University, East Lansing, Michigan; ‡ Yale School of Medicine, New Haven, Connecticut; § NYU Grossman School of Medicine, Department of Emergency Medicine, New York; || Yale School of Medicine, Department of Emergency Medicine, New Haven Connecticut (Previously at)

## Abstract

**Introduction:**

Medicine is increasingly influenced by politics, but physicians have historically had lower voter turnout than the general public. Turnout is even lower for younger voters. Little is known about the political interests, voting activity, or political action committee (PAC) involvement of emergency physicians in training. We evaluated EM trainees’ political priorities, use of and barriers to voting, and engagement with an emergency medicine (EM) PAC.

**Methods:**

Resident/medical student Emergency Medicine Residents’ Association members were emailed a survey between October–November 2018. Questions involved political priorities, perspective on single-payer healthcare, voting knowledge/behavior, and EM PACs participation. We analyzed data using descriptive statistics.

**Results:**

Survey participants included 1,241 fully responding medical students and residents, with a calculated response rate of 20%. The top three healthcare priorities were as follows: 1) high cost of healthcare/price transparency; 2) decreasing the number of uninsured; and 3) quality of health insurance. The top EM-specific issue was ED crowding and boarding. Most trainees (70%) were supportive of single-payer healthcare: “somewhat favor” (36%) and “strongly favor” (34%). Trainees had high rates of voting in presidential elections (89%) but less frequent use of other voting options: 54% absentee ballots; 56% voting in state primary races; and 38% early voting. Over half (66%) missed voting in prior elections, with work cited as the most frequent (70%) barrier. While overall, half of respondents (62%) reported awareness of EM PACs, only 4% of respondents had contributed.

**Conclusion:**

The high cost of healthcare was the top concern among EM trainees. Survey respondents had a high level of knowledge of absentee and early voting but less frequently used these options. Encouragement of early and absentee voting can improve voter turnout of EM trainees. Concerning EM PACs, there is significant room for membership growth. With improved knowledge of the political priorities of EM trainees, physician organizations and PACs can better engage future physicians.

## INTRODUCTION

The practice of medicine is increasingly influenced by politics. In the United States, tax-financed expenditures were estimated to fund 66% of all national health expenditures in 2020.^1^ Emergency medicine (EM) is particularly exposed to the effects of political changes because emergency departments see a disproportionately higher share of patients insured by Medicaid, a state- and federal government-funded program.^2^ Despite the important influence of elected officials on US healthcare, adjusted physician voter turnout rates have historically been lower than that of the general population.^3^,^4^ Recently, turnout has increased for physicians and is now similar to or slightly higher than the general population in the 2018 and 2020 elections.^5^ Voter turnout is still lower for millennials (the generation born 1981–1996), which includes most EM trainees, even though this demographic is quickly approaching the “baby boom” cohort (born 1946–1964) as the largest share of the electorate.^6^ While there are speculated reasons for low physician-voter turnout,^4^,^5^,^7^ less is known about trainees’ voting behaviors or barriers to voting.

Understanding the political priorities of future physicians is of critical importance for physician organizations and political action committees (PAC) in a time of partisan division. Health professional PACs have a significant monetary impact in election cycles; PACs contributed $24.9 million in the 2018 election cycle, surpassing the total amount from health insurers or hospital groups.^8^ However, EM trainees have low participation rates in the National Emergency Medicine PAC (NEMPAC),^9^ one of the largest EM PACS and the fourth largest contributor of all physician PACS, spending over $2 million in the 2018 election cycle.^10^ Despite high EM PAC contributions, EM trainee participation in an EM PAC is lower than other specialty physician trainees’ participation rates in their PACS.^11^–^13^ Little is currently known about the political interests of EM trainees, and highly engaged trainees in particular. Also less understood is the EM trainee’s perspective on specific key-item political topics such as single-payer health coverage, an issue of recurring interest to EM physician organizations.^14^

In this study our goal was to characterize EM trainees’ political priorities, knowledge, and experiences with voting as well as their participation and interest in EM PACs. We secondarily explored how political priorities vary by political party and voter registration varies by training level. We present data from EM residents and medical students who, as respondents to a survey from a trainee organization, are more likely to represent socially engaged individuals.^15^ As such, their political interests and PAC involvement have particular value to the institution of EM, as actively involved medical trainees are more likely to join physician organizations^16^ and make political campaign donations.^17^

## METHODS

### Study Design and Participants

This was a cross-sectional online survey emailed as an anonymous link to medical students and resident members of the Emergency Medicine Residents Association (EMRA) three times between October 1– November 16, 2018. The EMRA email list at the time of the survey comprised approximately 69% residents and 31% medical students. To recruit participants, we stratified trainees by training level (medical student vs resident); as part of a separate, unrelated study on survey incentives.^18^ The trainees were randomized to one of four incentive levels: one Amazon gift card worth $5, $25, $100, or none. Email subject lines were non-partisan as follows: “Planning to vote?”; “Make your voice heard”; and “Last call to participate!” The study was approved by the Yale institutional review board and is reported following the Strengthening the Reporting of Observational Studies in Epidemiology (STROBE) guidelines (see Appendix 3).^19^

**Population Health Research Capsule**

What do we already know about this issue?

*US politics influence medical practice, but physicians have lower voter turnout than the public. Less is known about EM trainees’ political priorities and behavior*.

What was the research question?

*We surveyed EM trainees on political priorities, voting behavior/barriers, and EM political action committee (PAC) use*.

What was the major finding of the study?

*Their top priority was the high cost of healthcare; 54% reported absentee ballots use and 38% reported early voting; just 4%, donated to EM PACs*.

How does this improve population health?

*Physician organizations can better engage trainees on their top issues of patient access. Early voting and absentee ballots will improve voter turnout for trainees*.

### Survey Outcomes

The survey was composed of three primary outcomes to describe EM trainee political priorities, voting, and PAC involvement (survey instrument in Appendix 2). The survey questions were informed by prior literature regarding medical trainees and their political interests and PAC involvement.^11^,^12^,^20^,^21^ In the first part of the survey, respondents were asked to rank their top three political priorities from the following subjects: 1) general healthcare; 2) emergency physician issues; and 3) US politics. We created this list of topics based on the current year’s National EMPAC 2018 candidate questionnaire, on Gallup Poll’s top issues for voters,^22^,^23^ the American College of Emergency Physicians’ Legislative & Regulatory Priorities, and this survey’s pilot feedback. As part of the political priorities section we also assessed opinions on single-payer healthcare, based on language used by the non-partisan Kaiser Family Foundation.^24^ In the second part of the survey, we assessed voting behavior and voting knowledge—registration, primaries, absentee, and early voting—based on questions from the US Census Voting and Registration Supplement.^25^ Lastly, we assessed participants’ awareness and engagement in any EM PAC.

### Survey Development

A 36-item survey covering political priorities, voting, and PACS was informed by published guidelines for questionnaire development.^26^ The survey was designed to take fewer than 10 minutes to complete. The authors who developed the survey included a health services researcher experienced in qualitative evaluations and two EM national representatives versed in EM trainee advocacy and health policy. To begin the survey development process, we searched relevant literature to assemble questions from existing surveys^11^,^12^,^20^–^25^ and, where necessary, developed new questions for the preliminary survey instrument. Using this initial survey, we conducted cognitive interviews with three EM residents to assess response process validity and ensure survey instrument comprehension. Interviewees verbalized their interpretation of the questions while taking the survey using the “think-aloud” approach. We then iteratively updated the survey following interviews if there was confusion on any questions. Next, pilot surveys were distributed to a convenience sample of eight trainees of different training levels and at institutions with geographical variety. The eight participants who completed the pilot survey provided written feedback on short forms following the survey. The survey was then edited in an iterative process to correct comprehension and technical issues based on the written pilot feedback and assessed to ensure outcomes were complete and appropriate.

### Survey Validity Approach

Validity evidence for our survey instrument is described following Messick’s sources of evidence framework adapted for medical education:^27^ 1) content: the wording of questions was derived from literature or developed with cognitive interviews and pilot feedback; 2) response process: respondents’ self-report of voting activity—an approach employed by the U.S. Census for national data on voting—-and political beliefs was conducted anonymously and thus less likely to be influenced by social desirability bias; 3) internal structure: where appropriate, variables were analyzed for reliability via Cronbach’s alpha, and theoretically related variables were assessed for correlations using Spearman’s correlation; and 4) relationships with other variables: comparing data to national data where possible.

### Data Analysis

We calculated response rates according to the American Association for Public Opinion Research (AAPOR) response rate 4 (RR4) definition.^28^ This calculation includes partial survey responses (AAPOR-defined as 50–80%) and completed surveys (AAPOR-defined as more than 80% complete), and considers a variable to estimate what proportion of cases of unknown eligibility are eligible. Because we did not know how many of the email addresses we included in the survey were active and, thus, what percentage was non-respondents vs potentially inactive email addresses, we estimated this eligibility variable by the maximum open rates of any previous email sent from the EMRA email list that year. We identified an open rate of 87% for students and 48% for residents and used these percentages for our eligibility variable. We evaluated non-response bias according to Halbesleben et al’s decision framework:^29^ a) wave analysis comparing the first to last respondents; b) comparing respondents to non-respondents based on available characteristics of gender, training year, and US Census Division; and c) comparing respondents to national benchmark data. We used frequency weighting to address differences between respondents and non-respondents on known characteristics from the whole population: gender; training year; and ZIP code.^30^

To determine aggregate ranking for political priorities, we scored choices following a Borda count approach^31^: 1^st^ = 3 points; 2^nd^ = 2 points, and 3^rd^ = 1 point. Standard descriptive statistics were used to report the primary outcome variables. We used chi-square tests to compare how demographics, political priorities, and single-payer perspective varied by political party, as well as how voter registration varied by training level with p<0.05 as the threshold for statistical significance. Data were included if at least 50% of the survey was completed and responses were dropped as missing if less than 50% was completed. We used Qualtrics LLC (Provo, UT) for survey management and Stata v16.1 (StataCorp LLC, College Station, TX) for analysis.

## RESULTS

### Participant Characteristics

**Figure 1:**
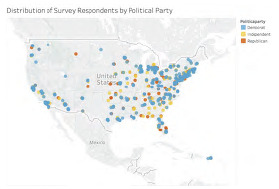
Geographic location in the United States of survey respondents based on ZIP code location. Location is displayed by the respondent’s stated political party.

Of 8,493 potential participants, the response rate calculated using the AAPOR RR4 definition was 20% including 1,241 individuals who completed 100% of the survey, 13 who completed over 80%, and 56 who partially completed between 50–80% of the survey. (See Appendix 1 for details.) Unless otherwise specified, proportions are reported below as unweighted for simplicity, given the similarities between weighted and unweighted results. Of the sample of 1,241 individuals, 500 were female (40%), and almost half were medical students (570/1271, 45% observed, 24% weighted) (Table 1). Most respondents of the observed sample were socially liberal (682/1241, 55%) and fiscally liberal (444/1232, 36%). Regarding political parties, weighted proportions were as follows: 47% Democrat, 29% Independent, and 11% Republican. Females, compared to males, were more likely to be Democrat (*P*<0.001), with 288/490 females (59%) reporting Democratic Party affiliation vs 348/741 males (47%) identifying as Democrats. The distribution of respondents was similar to the locations of EM residencies (eFigure 1), with the top areas being Middle Atlantic (22%) and East North Central (20%) (eTable 1). Respondents’ locations are displayed geographically by their reported political party in Figure 1.

### Survey Validity Assessment

In assessing the survey instrument’s reliability, Cronbach’s alpha comparing social and fiscal ideology scales (two items measuring conceptually similar outcomes) had acceptable internal consistency with α = 0.76. Supporting the survey’s construct validity, Spearman’s correlation indicated a significant association between theoretically similar groupings of being more liberal and Democrat and favoring single-payer healthcare coverage. Additionally, there was a significant correlation between awareness of absentee voting, early voting, and primaries (correlational matrix in eTable 2). While to our knowledge there is no national polling on political party identification for medical trainees, in comparing our survey findings to other published Gallup Poll national data, we found similarities between the increase in numbers of the Millennial generation identifying less frequently as Republicans and more frequently as Independents compared to older age categories.^32^ Compared to previously reported voting rates of residents from other specialties, 90% of plastic surgery trainees reported voting in the 2016 election compared to the 89% of EM trainees who reported in this survey as having voting in the 2016 election, although this is lower than national data for physicians (63%) in the 2018 election.^33^

### Non-response Bias Analysis

In addition to calculating frequency weighting for the responses based on gender, training year, and geographic location, we additionally conducted analyses to assess non-response bias. For this analysis, we followed a decision framework^29^ involving a wave analysis and comparison of respondents to non-respondents. We conducted a wave analysis comparing the first 200 respondents to the last 200 respondents based on their demographics and answers to survey questions on single-payer health insurance and PAC awareness (eTable 3). Late respondents had slightly lower rates of females (56% vs 66%, respectively), lower rates of medical students (30% vs 37%), were less likely to be from the South Atlantic region (12% vs 19%), more likely to be from the Pacific region (14% vs 8%). Late and early respondents were similar in political party, ideology, perspective on the issue of single-payer health insurance, and awareness of PACS. Next, we compared data between respondents and non-respondents based on known characteristics from the EMRA email list. Non-respondents compared to respondents had lower rates of medical students (27% vs 45%, respectively), and slightly lower rates of females (37% vs 40%) but similar geographic distribution (eTable 1). Lastly, compared to national data on emergency physician race and resident gender from the Association of American Medical Colleges in 2018, 34–36 (eTable 4) our study is similar to national data for female proportion (40% vs 36%, respectively), and representation of Black (5% vs 5%), and Hispanic (8% vs 5%).

### Political Priorities

#### General Healthcare Priorities

**Figure 2:**
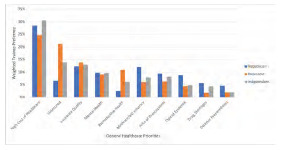
Weighted distribution of general healthcare priorities of trainees in emergency medicine. Participants ranked 1,2,3 level priority where 1 was highest concern and given 3 weighted points; level 3 priority was 1 point. The total points for each category were divided by total points per trainee grouping by party identification.

Overall, trainees ranked their top three healthcare priorities as follows: 1) high cost of healthcare/price transparency, 2) decreasing the number of uninsured, and 3) the quality of health insurance (Figure 2, tabular form in eTable 5) .The rest of the priorities were ranked in the following order: mental health services availability: family planning/women’s reproductive health; Medicare/Medicaid solvency for the future; high cost of prescriptions; the opioid epidemic; drug shortages; and disaster preparedness. The ranking of priorities differed significantly by political party affiliation across all three sets of issues: general healthcare; emergency physician issues; and general politics (*P*<0.05). The most considerable differences in rank were as follows: a) decreasing the uninsured was ranked second by both Independents and Democrats compared to seventh by Republicans; b) reproductive healthcare was ranked fourth for Democrats compared to seventh for Independents and 10^th^ for Republicans; and c) solvency for Medicare and Medicaid was ranked third for Republicans compared to seventh for Democrats, and sixth for Independents.

#### Emergency Physician Priorities

**Figure 3:**
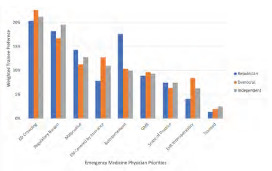
Weighted distribution of emergency physician priorities of EM trainees in emergency medicine. Participants ranked 1,2,3 level priority where 1 was the highest concern and given three weighted points, level 3 priority was 1 point. The total points for each category were divided by total points per trainee grouping by party identification.

For emergency physician-specific issues, the top concerns were as follows: 1) ED crowding and boarding; 2) regulatory burden on physicians; and 3) malpractice reform. (Figure 3, tabular form in eTable 6). The rest of the priorities were ranked in the following order: emergency services as a covered insurance benefit; physician reimbursement; federal funds for graduate medical education residency slots; the scope of practice (physician supervision of advanced practice practitioners), health information exchange interoperability; and telemedicine and other modern delivery systems. There were similarities in ranking across political party affiliation among the top three issues for this category. There were differences by political party affiliation for some lower rated matters: a) reimbursement was third for Republicans and fifth for Democrats and Republicans; and b) EM services covered by insurance was third and fourth for Democrats and Independents, respectively, but was sixth for Republicans.

#### American Political Priorities

For general American political issues, the priorities were as follows: 1) healthcare; 2) wealth inequality; and 3) education (eFigure 2, tabular form in eTable 7). The rest of the priorities were ranked in the following order: political corruption; racial disparities; federal budget deficit/spending/taxes; environment/pollution; gun safety/control; economy/unemployment/jobs; immigration; foreign policy; national security; criminal justice reform; and drug policy. Although healthcare was a top issue for all political parties responding, priorities varied widely by political party. The most considerable differences by top priorities were a) wealth inequality was second for Democrats, third for Independents, and 12^th^ for Republicans; b) racial disparities were third for Democrats, sixth for Independents, and 11^th^ for Republicans; and c) the budget deficit was first for Republicans, fifth for Independents, and eighth for Democrats.

**Figure 4:**
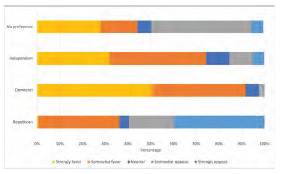
Weighted distribution of emergency medicine trainees’ opinions on single-payer healthcare by political party identification *EM*, emergency medicine.

Regarding opinions on single-payer insurance, overall, trainees were highly supportive (869/1239, 70%: “somewhat” (36%), and “strongly favor” (34%) (Figure 4) Opinions on single-payer insurance differed significantly by party lines (*P*<0.05), with most Democrats (564/637, 89%) in favor of it and the majority of Republicans (126/176, 72%) against.

#### Voting

**Figure 5:**
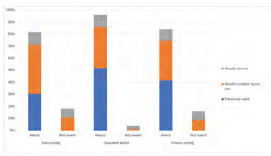
Weighted distribution of emergency medicine trainees’ voting knowledge and use of voting options.

Trainees reported high rates of Election Day voting but lower use of early voting opportunities or absentee ballot (Figure 5). Most respondents (89% (1043/1170)) reported voting in the last presidential election. While most respondents reported awareness of absentee voting, early voting, and primary elections (96%, 84%, and 90%, respectively), of those who were aware, far fewer reported previously using absentee ballots (644/1192, 54%), early voting (399/1038, 38%), and voting in state primaries (619/1104, 56%). Of those who had not previously voted early, absentee, or in the primaries, approximately one-third to half would want to learn more or consider future use of these voting options.

Many trainees did not vote due to commonly cited barriers (eFigure 3). More than half (66%, 771/1169) of EM trainees had missed voting in prior elections. Among those who missed voting or had never voted, common reasons were working (525/752, 70%); personal life (374/737, 51%); didn’t feel voting made a difference (304/738, 41%); forgot to vote (294/723, 41%); and failed to register (182/711, 26%). Free-text responses also cited travel, being out of their home district, forgetting to request or send in absentee ballots, or not knowing enough about the candidates.

Approximately a quarter of trainees had voter registration at a previous address (300/1169, 26%). Registration at an earlier address varied significantly (*P*<0.01) by training level such that medical students (MS) and residents in their first postgraduate year (PGY) had almost double the rate of still being registered at a previous address compared to those in their fourth year: medical students’ previous address registrations were MS1 (43%, 6/14) vs MS4 (21%, 76/285); resident previous address registrations were PGY1 (40%, 86/214) vs PGY4 (17 %, 13/77).

#### Political Action Committee

Engagement in the PAC was low, but many respondents were interested in learning more (eFigure 4). Just over half (767/1238, 62%) of trainees knew there was an EM PAC. Only 7% (52/767) of those who were aware had contributed (4% of 1,238 respondents to the PAC awareness question). Equal proportions of EM trainees who reported they were aware of the PAC would consider donating (50%) 380/767 or were not interested (44%) 335/767. Many free-text respondents who wanted to avoid contributing to the PAC reported financial difficulties as a barrier.

## DISCUSSION

To our knowledge, this is the first national study to investigate EM trainees’ political awareness, interests, and behavior. Importantly, their main general healthcare priorities centered around the affordability of healthcare and insurance coverage. Regarding EM-specific priorities, ED crowding and boarding were top concerns, while reimbursement was a lower priority.

These rankings differ substantially from that of other specialty physicians’ prioritization: In a 2014 study of 397 young plastic surgery physicians, the scope of practice and Medicare reimbursement were first and second highest priority concerns by 277 and 202 respondents, respectively, while the patient access issue, the “Patient Protection/Affordable Care Act,” was ranked as seventh level priority. 20 In a 2009 study of 2,689 young surgeons, reimbursement was the top concern.^21^ These differences may reflect the older age groups surveyed in those studies—most were between the ages 30–40—or may reflect the differences in preferences between surgeons, who have higher proportions of physicians who contribute to Republican candidates,^37^ and emergency physicians.

Recent national surveys of medical students have also reflected their high concerns for patients’ ability to afford care.^38^ These concerns about the cost of healthcare and access issues may explain our survey’s demonstration of the strong support for single-payer health coverage. Our finding that 70% of trainees support single-payer coverage aligns with a 2007 national poll that reported a similar level of support by practicing emergency physicians.^39^

Additionally, recent events of medical student mobilization for single-payer advocacy within the American Medical Association further reflect the importance of this issue to medical trainees.^40^ Moreover, the preponderance of EM trainees identifying as Democratic/Independent mirrors the trend in medicine of a shift from the previous conservative base.^37^,^41^ This liberalization may be due partly to generational shifts,^32^ the increasing number of females in medicine^37^ and EM,^42^ employee status vs independent practice,^37^ and the influence of student debt.

Concerning voting, many trainees cited work commitments as a barrier. This finding is consistent with previous national surveys of US citizens, which have shown that practicing physicians are more likely than the general public to cite not voting due to being “too busy, conflicting work or school.”^4^ However, in contrast to practicing physicians who showed an increased likelihood to vote early,^4^ EM trainees had low early voting use (38%) but high interest in early voting in future elections (47%). Additional barriers trainees in our study included forgetting to vote or not having a current registration, issues which may be amenable to institutional support and initiatives. These initiatives include flexible Election Day scheduling to allow trainees to vote, reminders to vote early or register for an absentee ballot, and voter registration campaigns, which should be targeted to trainees upon relocating to a new institution, such as Citizen Physicians and TurboVote. Increased visibility and recognition of the importance of voting by medical trainee governing bodies such as the Association of American Medical Colleges and the Accreditation Council for Graduate Medical Education could legitimize Election Day scheduling adjustments for trainees to get to the polls.

Additionally, this is the first publicly reported survey of EM trainees’ interest and participation in an EM PAC. Despite high election voting rates and reasonable awareness of the EM PAC (62%), respondents had low rates of contributing (4%). Notably, half of all trainees already aware of the PAC (50%) would “consider donating” if given more information, indicating room for potential growth in PAC awareness and membership with proper outreach and messaging. As consolidation in hospitals rises both hospital prices^43^ and costs to patients^44^ without a commensurate rise in physician prices,^45^ it will become increasingly crucial for PACs to champion causes that matter to individual physicians, especially as physicians increasingly become employees of large practice groups.^46^,^47^ To fortify the pipeline of contributors, PACs should focus on membership development of trainees and articulating ways in which PAC goals align with trainees’ top political priorities.

## LIMITATIONS

This study has limitations. First, the 20% response rate can be interpreted to limit the generalizability of the findings to non-respondent EM trainees. However, studies of response rates have found that lower responses are less inherently a sign of nonrepresentative data than previously assumed.^48^,^49^ Additionally, wave analysis revealed only slight differences between early to late responders, and national demographics were similar to survey demographics. Moreover, survey respondents’ geographical distributions were similar to national EM residency programs, and respondents were of similar race/ethnicity and gender proportions compared to national data. While these analyses and literature are reassuring, we acknowledge that this survey’s findings are likely representative of a trainee who is more likely to be civically engaged than a non-respondent, as suggested by higher-than-expected voting rates.

Although this possible response bias may limit assumptions on the whole of EM trainees, we feel knowledge of this group’s interests are especially important because EM trainees who are engaged now are more likely to be involved in physician organizations and advocacy in the future. Secondly, the reliance on self-report of political activity may limit the internal validity and could contribute to why trainees had high voting rates. Lastly, as a quantitative study, we could not gain a more detailed understanding of political priorities than ranking from pre-specified lists, which limits knowledge of alternative preferences. Future research could use qualitative methods to explain political priorities further.

## CONCLUSION

Physicians’ participation in the political conversation is even more critical as the US continues to face challenges at the intersection of politics and healthcare. Trainees in EM prioritized healthcare access issues, including the cost of care, health insurance quality, and ED boarding. Single-payer health insurance was favored by most respondents. Many EM trainees reported high voting levels but lower use of early or absentee voting and lower financial contributions to EM political action committees. With more at stake in the political process, it is even more urgent that the house of medicine prioritizes efforts to recruit, train, and retain future healthcare advocates.

## Supplementary Information



## Figures and Tables

**Table 1:** Characteristics of emergency medicine trainee respondents.

Overall	Non-response weighted %							
	Observed N (1,241)	Observed %	Non-response weighted %	Republican (11.3%)	Democrat (46.9%)	Independent (28.5%)	No preference (13.3%)	P-value
Gender								
Female	500	40.3%	40.1%	11.3%	54.5%	27.8%	6.4%	<0.001
Male	741	59.7%	59.9%	11.3%	41.8%	29.1%	17.9%	
Training year								
M1–2	82	6.1%	4.3%	13.7%	45.5%	32.2%	8.3%	<0.001
M3	106	8.5%	5.0%	6.7%	50.2%	31.2%	11.7%	
M4	382	30.8%	14.9%	12.7%	53.5%	27.5%	6.3%	
PGY1	231	18.6%	26.3%	14.0%	56.2%	25.2%	4.6%	
PGY2	189	15.2%	20.7%	4.6%	38.8%	31.8%	24.7%	
PGY3	155	12.5%	18.6%	9.3%	37.5%	29.2%	24.1%	
PGY4	86	6.9%	10.0%	20.6%	46.6%	27.9%	4.8%	
Missing	10	0.8%	0.2%	12.5%	6.3%	25.0%	56.3%	
Race								
White	956	77.0%	75.6%	12.0%	45.6%	29.7%	12.7%	<0.001
Black	57	4.6%	3.0%	1.2%	66.4%	22.4%	10.0%	
Asian	201	16.2%	18.2%	11.0%	44.7%	26.2%	15.6%	
American Indian	9	0.7%	0.3%	31.0%	27.6%	27.6%	13.8%	
Native Hawaiian/Pacific Islander	4	0.3%	0.1%	45.4%	0.0%	0.0%	54.6%	
Missing	14	1.1%	2.8%	0.0%	64.2%	20.6%	15.2%	
Ethnicity								
Hispanic/Latino	99	8.0%	6.3%	20.0%	55.7%	7.6%	16.7%	<0.001
Not Hispanic/Latino	1122	90.1%	92.2%	10.7%	44.7%	29.3%	13.1%	
Missing	20	1.6%	1.5%	10.9%	14.7%	68.2%	6.2%	
SOCIAL political ideology								
Extremely liberal	171	13.8%	11.0%	0.8%	92.8%	4.5%	1.9%	<0.001
Liberal	682	55.0%	57.9%	5.6%	50.6%	37.6%	6.2%	
Neutral	152	12.3%	11.5%	22.3%	9.1%	26.7%	41.5%	
Conservative	144	11.6%	11.4%	42.8%	6.7%	17.0%	33.5%	
Extremely conservative	16	1.3%	0.7%	77.2%	10.5%	12.3%	0.0%	
Missing	76	6.1%	7.5%	0.2%	73.1%	16.2%	10.5%	
FISCAL political ideology								
Extremely liberal	38	3.1%	2.4%	0.0%	91.8%	8.2%	0.0%	<0.001
Liberal	444	35.8%	32.5%	0.1%	76.2%	19.2%	4.5%	
Neutral	275	22.2%	22.6%	10.5%	55.3%	25.2%	9.0%	
Conservative	382	30.8%	33.0%	16.9%	10.1%	46.2%	26.9%	
Extremely conservative	59	4.8%	4.4%	75.4%	7.9%	9.7%	7.1%	
Missing	34	3.5%	5.1%	0.0%	74.0%	14.8%	11.2%	

Note: Two-sided P-values taken from chi-squared tests of non-response weighted values. Non-response weights based on gender, training year, and geographic location.

*PGY*, postgraduate year.
