# Supplementary material for: Political Priorities, Voting, and Political Action Committee Engagement of Emergency Medicine Trainees: A National Survey
Source: West J Emerg Med. 2023 May 5;24(3):469–78. doi: 10.5811/westjem.59351 (PMC10284518; doi:10.5811/westjem.59351)
Supplement: Supplementary file 1 [file wjem-24-469-Appendix_1-3.docx]

Online Supplement:

Political Priorities, Voting, and Political Action Committee Engagement of Emergency Medicine Trainees: A National Survey

[Appendix 1. AAPOR Survey Calculation 3](#_Toc118485238)

[eTable 1. Demographics of respondents versus non-respondents 4](#_Toc118485239)

[eFigure 1. Survey Respondents Distribution Compared to EM Programs locations 5](#_Toc118485240)

[eTable2. Construct Validity Correlation Matrices 6](#_Toc118485241)

[eTable 3. Nonresponse Bias Analysis: Wave Analysis for Early to Late Respondents 7](#_Toc118485242)

[eTable 4. Nonresponse Bias Analysis: National EM Demographic Data 9](#_Toc118485243)

[eTable 5. General Healthcare Priorities as Ranked by EM Trainees, by Political Party 10](#_Toc118485244)

[eTable 6. EM Physician Priorities as Ranked by EM Trainees, by Political Party 11](#_Toc118485245)

[eFigure 2. American Political Priorities as Ranked by EM Trainees, by Political Party 12](#_Toc118485246)

[eTable 7. American Political Priorities as Ranked by EM Trainees, by Political Party 13](#_Toc118485247)

[eFigure 3. Weighted Distribution of EM Trainees Cited Barriers to Voting, by Training Level 14](#_Toc118485248)

[eFigure 4. Weighted Distribution of EM Trainees Awareness and Participation with an EM PAC 15](#_Toc118485249)

[Appendix 2: Survey Instrument 16](#_Toc118485250)

[Appendix 3: STROBE Checklist 36](#_Toc118485251)

# Appendix 1. AAPOR Survey Calculation

|  | 1241 | **I** = Complete interview (1.1) |
| --- | --- | --- |
|  | 56 | **P** = Partial interview (1.2) |
|  | 90 | **R** = Refusal and break-off (2.10) |
|  | 0 | **NC** = Non-contact (2.20) |
|  | 0 | **O** = Other (2.30) |
| 0.31*8493 | 2599 | **UO** = Unknown, other **(student)** |
| 0.69*8493 | 5893 | **UO** = Unknown, other **(resident)** |
|  | 0.87 | **e** = Estimated proportion **(student)** |
|  | 0.47 | **e** = Estimated proportion **(resident)** |


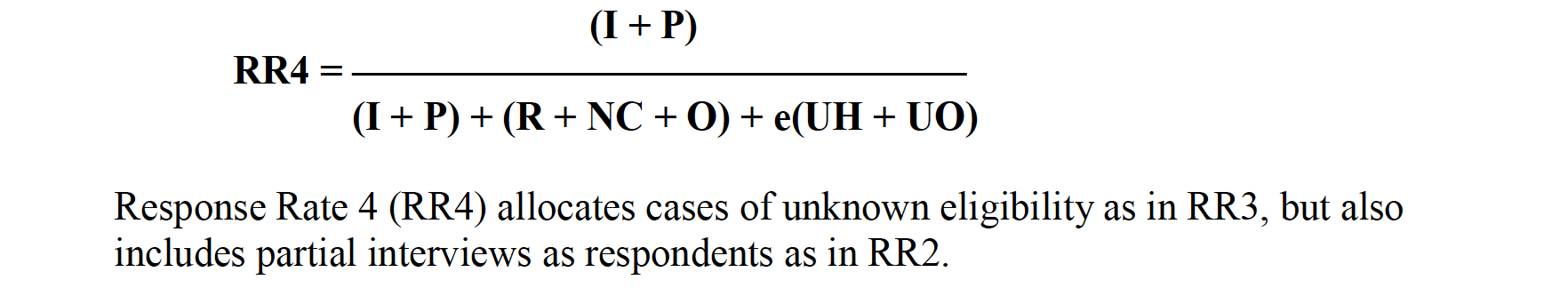


**RR4= (1254+56)/ ((1254+56) +(90+0+0) + .87(2599) + .47(5893))= 20.3%**

In the above calculation, our total email send was 8493 individuals. Based on the EMRA member list, we know that 69% of these are residents, and 31% of these are students. We consider e ( estimated proportion) to be the maximum open rate for previous EMRA email list sends, of which in October 2018, the “EMRA What’s Up” email, 87% of students opened the email and 48% of residents opened the email.

# eTable 1. Demographics of respondents versus non-respondents

|  | **All EMRA** | | **Responders** | | **Non-responders** | |
| --- | --- | --- | --- | --- | --- | --- |
| **Training year*** |  |  |  |  |  |  |
| Medical student | 2,522 | 29.7% | 570 | 46.3% | 1,952 | 26.9% |
| Resident/Fellow | 5,971 | 70.3% | 661 | 53.7% | 5,310 | 73.1% |
| **Gender** |  |  |  |  |  |  |
| Female | 3,176 | 37.4% | 500 | 40.3% | 2,676 | 36.9% |
| Male | 5,317 | 62.6% | 741 | 59.7% | 4,576 | 63.1% |
| **US Census Division** |  |  |  |  |  |  |
| Pacific | 909 | 10.7% | 134 | 10.8% | 775 | 10.7% |
| Mountain | 429 | 5.1% | 66 | 5.3% | 363 | 5.0% |
| West North Central | 461 | 5.4% | 78 | 6.3% | 383 | 5.3% |
| East North Central | 1,664 | 19.6% | 247 | 19.9% | 1,417 | 19.5% |
| West South Central | 831 | 9.8% | 88 | 7.1% | 743 | 10.2% |
| East South Central | 335 | 3.9% | 51 | 4.1% | 283 | 3.9% |
| South Atlantic | 1,485 | 17.5% | 216 | 17.4% | 1,270 | 17.5% |
| Middle Atlantic | 1,857 | 21.9% | 277 | 22.4% | 1,579 | 21.8% |
| New England | 523 | 6.2% | 85 | 6.8% | 438 | 6.0% |
|  |  |  |  |  |  |  |
| * Limited in all known data to categorization by medical student, resident, or fellow membership type | | | | | | |

# eFigure 1. Survey Respondents Distribution Compared to EM Programs locations


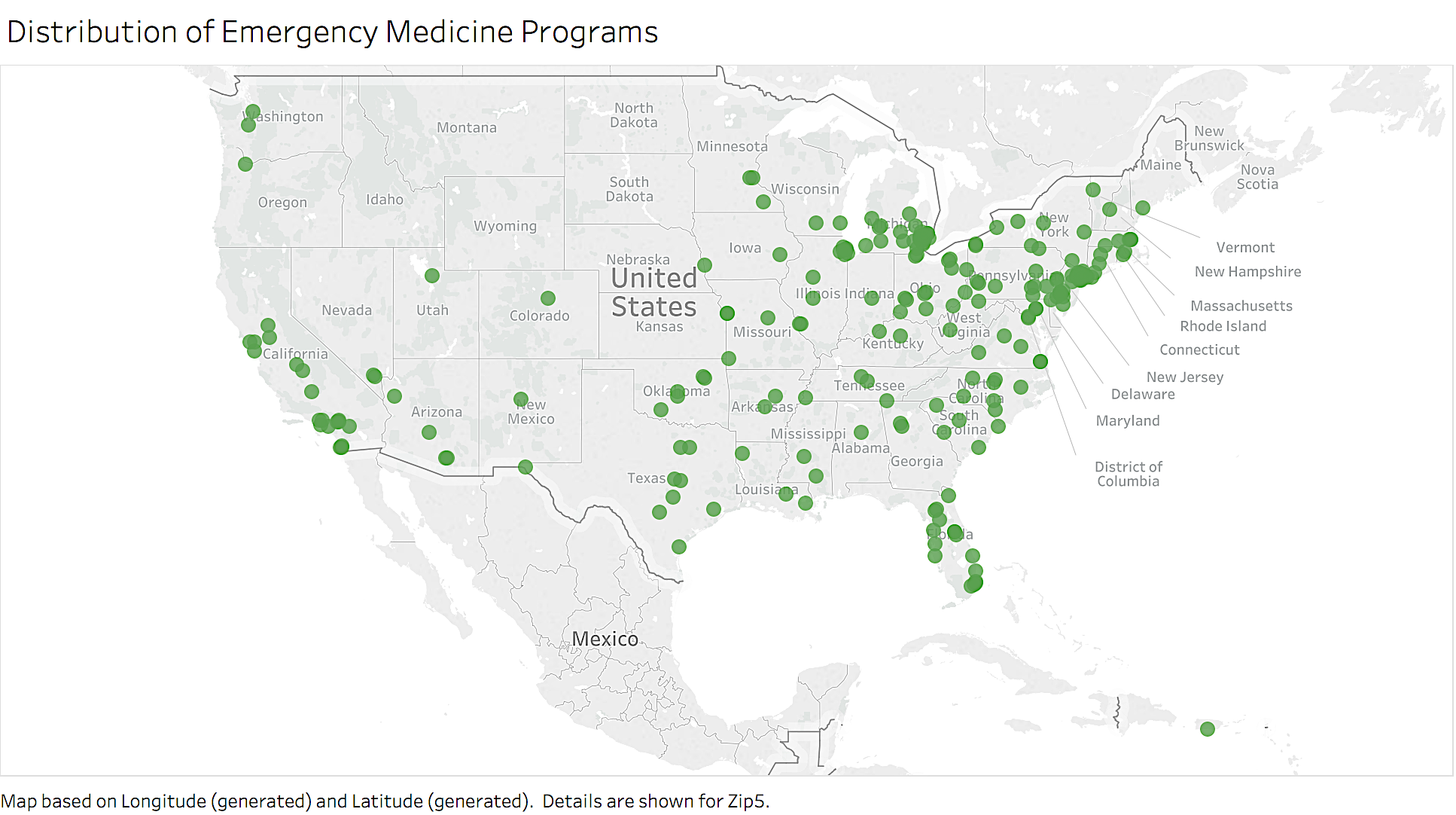


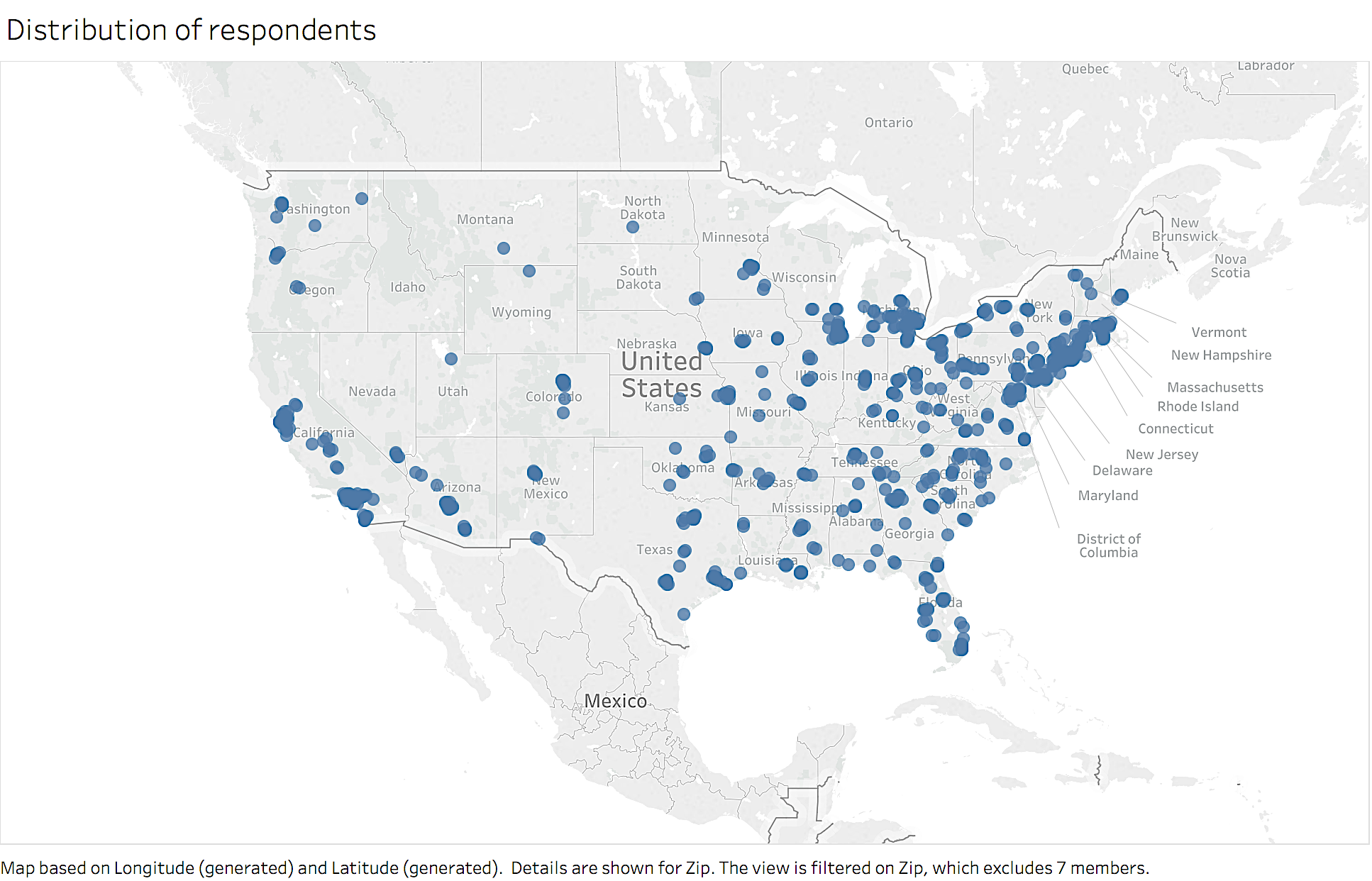


# eTable2. Construct Validity Correlation Matrices

| **Reported political affiliations and policy perspective** | Democrat | More Liberal | Favor Single-Payer |
| --- | --- | --- | --- |
| Democrat | 1 |  |  |
| More Liberal | 0.4592* | 1 |  |
| Favor Single-Payer | 0.3577* | 0.2897* | 1 |

| **Reported Awareness** | Absentee | Early Voting | Primaries |
| --- | --- | --- | --- |
| Absentee | 1 |  |  |
| Early Voting | 0.2445* | 1 |  |
| Primaries | 0.3461* | 0.2959* | 1 |

(*) denotes significance at below 0.5

­­

# eTable 3. Nonresponse Bias Analysis: Wave Analysis for Early to Late Respondents

|  | **Early respondents** | **Late respondents** |
| --- | --- | --- |
|  | First 200 responses | Last 200 responses |
| **Gender** |  |  |
| Male | 34.0% | 44.0% |
| Female | 66.0% | 56.0% |
| **Training year** |  |  |
| M1-2 | 25.5% | 25.0% |
| M3 | 10.0% | 7.5% |
| M4 | 37.0% | 29.5% |
| PGY1 | 10.5% | 17.0% |
| PGY2 | 13.5% | 11.5% |
| PGY3 | 3.0% | 9.0% |
| PGY4 | -- | -- |
| Unknown | 0.5% | 0.5% |
| **US Census Division** | |  |
| Pacific | 8.3% | 13.7% |
| Mountain | 6.2% | 7.4% |
| West North Central | 7.3% | 7.4% |
| East North Central | 18.7% | 21.6% |
| West South Central | 6.7% | 3.7% |
| East South Central | 3.1% | 3.7% |
| South Atlantic | 19.2% | 12.1% |
| Middle Atlantic | 24.4% | 24.2% |
| New England | 6.2% | 6.3% |
| **Political affiliation** |  |  |
| Democrat | 51.5% | 53.0% |
| Independent | 26.5% | 20.0% |
| Republication | 13.0% | 12.5% |
| Other | 2.0% | 4.5% |
| No preference | 7.0% | 10.0% |
| **Social political ideology** | |  |
| Extremely liberal | 14.6% | 15.9% |
| Liberal | 31.3% | 33.3% |
| Neutral | 25.5% | 25.7% |
| Conservative | 15.6% | 13.1% |
| Extremely conservative | 9.9% | 7.7% |
| Unknown | 3.1% | 4.4% |
| **Fiscal political ideology** | |  |
| Extremely liberal | 3.6% | 2.1% |
| Liberal | 13.0% | 18.6% |
| Neutral | 26.9% | 27.3% |
| Conservative | 19.2% | 21.7% |
| Extremely conservative | 23.3% | 18.6% |
| Unknown | 1.6% | 5.2% |
| **Favor single-payer system** | |  |
| Strongly favor | 34.0% | 36.5% |
| Somewhat favor | 39.0% | 35.5% |
| Neutral | 6.0% | 11.0% |
| Somewhat oppose | 12.5% | 8.0% |
| Strongly oppose | 8.5% | 9.0% |
| **Aware of EM-PACs** |  |  |
| No | 38.0% | 42.5% |
| Yes | 62.0% | 57.5% |

# eTable 4. Nonresponse Bias Analysis: National EM Demographic Data

| Data assembled from AAMC Tables available from:  EM Females : https://www.aamc.org/data-reports/workforce/data/table-12-practice-specialty-females-race/ethnicity-2018 |
| --- |
| EM Males: https://www.aamc.org/data-reports/workforce/data/table-12-practice-specialty-females-race/ethnicity-2018 |

# eTable 5. General Healthcare Priorities as Ranked by EM Trainees, by Political Party

# eTable 6. EM Physician Priorities as Ranked by EM Trainees, by Political Party

# eFigure 2. American Political Priorities as Ranked by EM Trainees, by Political Party


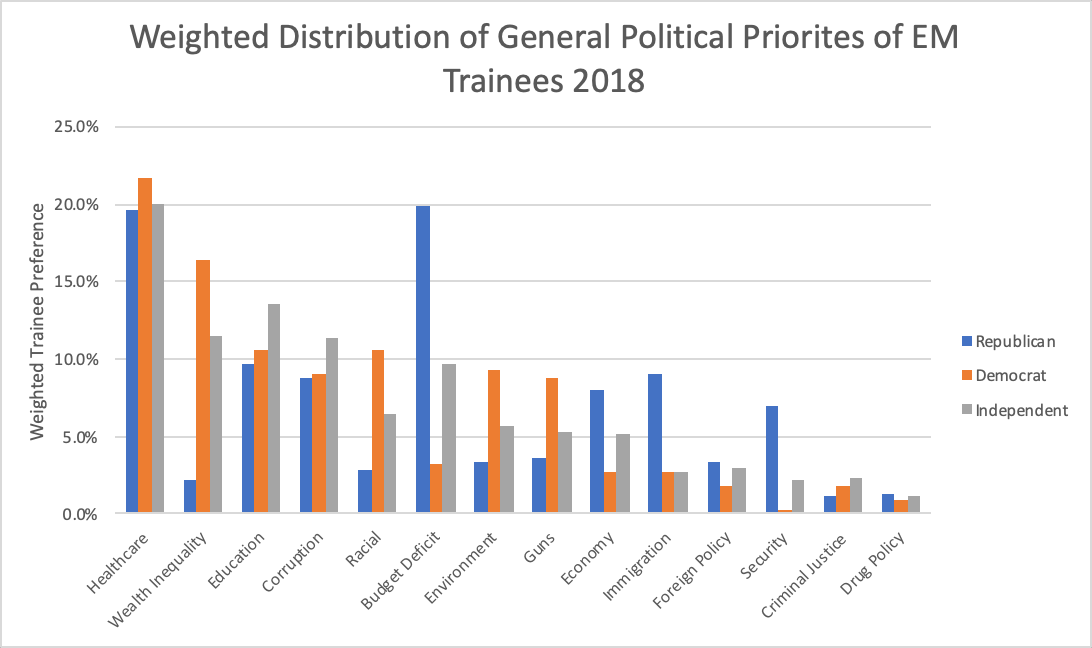


*eFigure 2. Participants ranked 1,2,3 level priorities where 1 was highest concern and given 3 weighted points, level 3 priority was 1 point. The total points for each category was divided by total points per trainee grouping by party identification.*

# eTable 7. American Political Priorities as Ranked by EM Trainees, by Political Party

#

# eFigure 3. Weighted Distribution of EM Trainees Cited Barriers to Voting, by Training Level


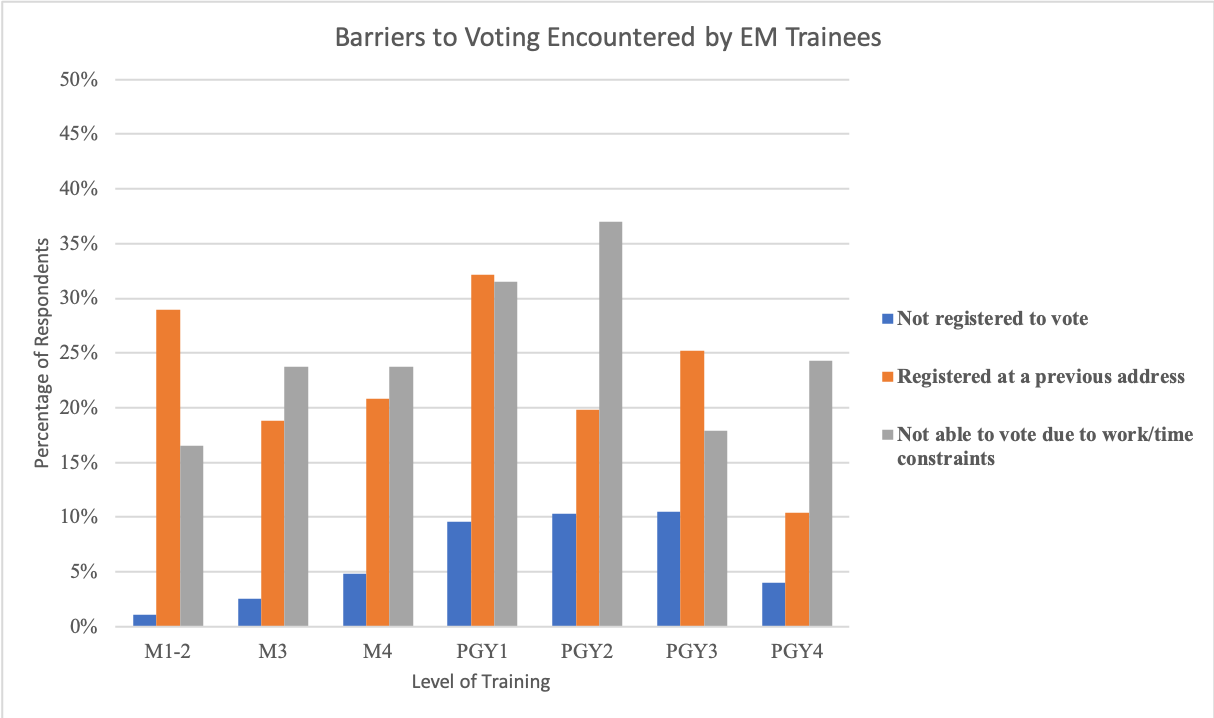


# eFigure 4. Weighted Distribution of EM Trainees Awareness and Participation with an EM PAC


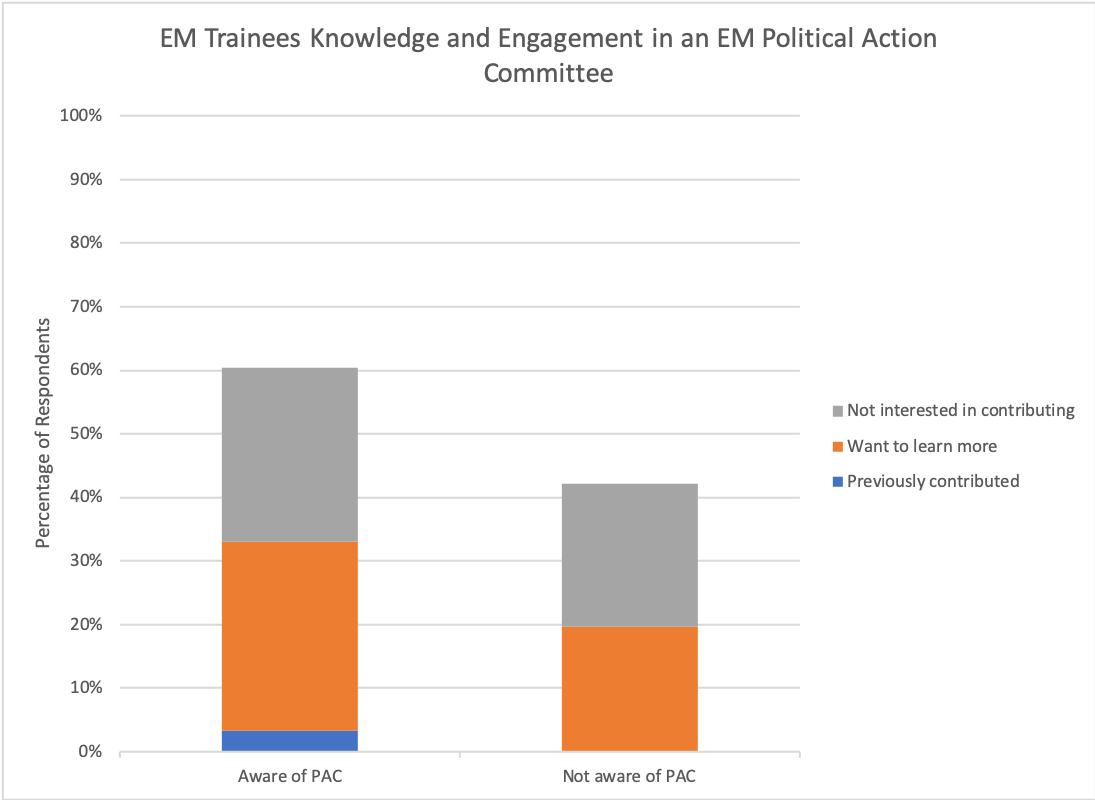


# Appendix 2: Survey Instrument

Standard: Block 2 (2 Questions)

Block: General demographics (7 Questions)

Standard: Demographics Political (5 Questions)

Standard: Interests (4 Questions)

Standard: Voting habits (10 Questions)

Standard: Early/ Absentee/ Primaries (7 Questions)

Standard: NEMPAC (5 Questions)

Standard: Pledge (3 Questions)

| Page Break |  |
| --- | --- |

Start of Block: Block 2

Q1


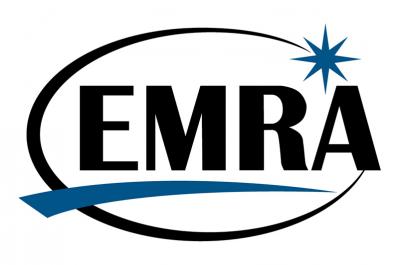


Q2 We are a team of researchers from <<blinded for review>> conducting a study to examine the political behavior and interests of emergency medicine residents and students. You are invited to participate in a survey that will take approximately 5 minutes. You will be asked questions on your political viewpoints, past political behavior, and demographics.  There are no anticipated risks for participating. Your participation in this study is completely voluntary, and you are free to refuse to answer any questions or you may choose to end your participation at any time. Answers will be kept confidential.  Your decision of whether or not to participate will not affect your relationship with EMRA. If you have concerns, complaints, or questions, contact <<blinded for review>> Human Subjects Committee, 203-785-4688, Human.Subjects@<<blinded for review>> edu. If you would like to participate, simply check "yes" to start the survey.

- Yes (1)
- No (2)

Skip To: End of Survey If Q2 = 2

End of Block: Block 2

Start of Block: General demographics

| 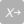 | 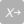 |
| --- | --- |

Q3 What race do you most identify with? Please choose one:

- White (1)
- Black or African American (2)
- Asian (3)
- Native Hawaiian or other Pacific Islander (4)
- American Indian or Alaska Native (5)

| 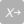 |
| --- |

Q4 Are you of Hispanic or Latino descent?

- Hispanic or Latino (1)
- Not Hispanic or Latino (0)

Q5 What is your gender:

- Male (1)
- Female (2)
- Other (4) ________________________________________________

Q6 Please select your current training level:

▼ MS1 (11) ... Other (10)

Q7 What is your zip code? (5 digit)

________________________________________________________________

Q8 What is your name? First Last *(This helps us ensure the survey is not taken multiple times. Answers will be kept confidential)*

________________________________________________________________

Q9 What is your email?

________________________________________________________________

| Page Break |  |
| --- | --- |

End of Block: General demographics

Start of Block: Demographics Political

| 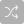 |
| --- |

Q10 What is your current political affiliation?

- Republican (1)
- Democrat (2)
- Independent (3)
- Other (4) ________________________________________________
- No preference (5)

Display This Question:

If Q10 = 1

Q11 Would you call yourself a strong Republican or a not very strong Republican?

- Strong (1)
- Not very strong (2)

Display This Question:

If Q10 = 2

Q12 Would you call yourself a strong Democrat or a not very strong Democrat?

- Strong (1)
- Not very strong (2)

Q13 Here is a 7-point scale on which the political views that people might hold are arranged from extremely liberal (left) to extremely conservative (right). Where would you place yourself on this scale in terms of SOCIAL issues?

|  | Extremely liberal | Extremely conservative |
| --- | --- | --- |

|  | 1 | 2 | 3 | 4 | 5 | 6 | 7 |
| --- | --- | --- | --- | --- | --- | --- | --- |

| Political Ideology () | 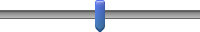 |
| --- | --- |

Q14 Here is a 7-point scale on which the political views that people might hold are arranged from extremely liberal (left) to extremely conservative (right). Where would you place yourself on this scale in terms of FISCAL issues?

|  | Extremely liberal | Extremely conservative |
| --- | --- | --- |

|  | 1 | 2 | 3 | 4 | 5 | 6 | 7 |
| --- | --- | --- | --- | --- | --- | --- | --- |

| Political Ideology () | 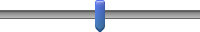 |
| --- | --- |

| Page Break |  |
| --- | --- |

End of Block: Demographics Political

Start of Block: Interests

| 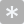 | 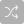 |
| --- | --- |

Q15 Select the top THREE health care issues you care most about:
(Write in 1, 2, 3 with 1 being a topic you care MOST about)

______ Quality of insurance (pre-existing conditions, no lifetime limits) (1)

______ Decreasing the number of uninsured (18)

______ Medicare/Medicaid solvency for the future (4)

______ High cost of prescription drugs (6)

______ Mental health services availability (7)

______ Opioid epidemic (13)

______ Family planning/ women's reproductive health (16)

______ High cost of healthcare/ price transparency (23)

______ Drug shortages (24)

______ Disaster preparedness (26)

______ Other (19)

| Page Break |  |
| --- | --- |

| 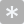 | 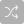 |
| --- | --- |

Q16 These issues may be of particular interest to physicians. 
Select the top THREE issues you care most about:
(Write in 1, 2, 3 with 1 being a topic you care MOST about)

______ Telemedicine and other modern delivery systems (7)

______ Federal funds for GME residency slots (8)

______ Malpractice reform (9)

______ Scope of practice (physician supervision of advanced practice providers) (10)

______ Regulatory burden on physicians (15)

______ ED crowding and boarding (20)

______ Health information exchange interoperability (22)

______ Physician reimbursement (25)

______ Emergency services as a covered insurance benefit (28)

______ Other (19)

| Page Break |  |
| --- | --- |

| 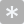 | 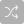 |
| --- | --- |

Q17 What do you think are the top THREE most important issues facing the country today?
(Write in 1, 2, and 3, with 1 being a topic you care most about)

______ Health care (1)

______ Economy, unemployment, jobs (2)

______ Federal budget deficit, spending, taxes (3)

______ Immigration (4)

______ Gun safety/ gun control (5)

______ Education (6)

______ Environment/ Pollution (7)

______ National security (8)

______ Racial disparities (9)

______ Political corruption (12)

______ Wealth inequality (11)

______ Foreign policy (10)

______ Criminal justice reform (14)

______ Drug policy (15)

______ Other (13)

| Page Break |  |
| --- | --- |

Q18 Do you favor or oppose having a national health plan, or a single-payer plan, in which all Americans would get their insurance from a single government plan?

- Strongly favor (1)
- Somewhat favor (2)
- Somewhat oppose (3)
- Strongly oppose (4)
- Don't know (5)

| Page Break |  |
| --- | --- |

End of Block: Interests

Start of Block: Voting habits

Q19 Are you registered to vote?

- Yes (1)
- No (2)
- I don't know (4)

Display This Question:

If Q19 = 1

Q20 Is your voter registration for your current address?

- Yes, my voter registration is for my current address (1)
- No, my voter registration is for my previous address (2)

Display This Question:

If Q19 = 2

And Q19 = 4

Q21 If you are not currently registered to vote, select which statement applies:

- I have never registered to vote (1)
- I have registered in the past, but not for my current address (2)
- I don't know (4)

Display This Question:

If Q20 = 2

Or Q19 = 2

Or Q19 = 4

Q22 Do you plan to register to vote by October 1st 2018? *(For some states this is the deadline to register in order to vote in the November 6th elections.)*

- Yes (1)
- No (2)

| Page Break |  |
| --- | --- |

Q23 Which of the following elections have you previously voted in?

- State/ local elections (2)
- Mid-term elections (3)
- Presidential elections (4)
- I am not sure (5)
- I have never voted (1)

Display This Question:

If Q23 != 1

Q24 Did you vote in the last presidential election?

- Yes (1)
- No (2)
- I don't remember (3)

Display This Question:

If Q23 != 1

Q25 Have you ever missed voting in an election in which you were able to vote?

- No, I have voted in every single election every year (1)
- Yes, I have missed voting some election year(s) (2)

Display This Question:

If Q25 = 2

Or Q23 = 1

| 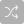 |
| --- |

Q26 If you missed voting in an election, what was your reason for not voting? 

|  | Disagree (1) | Agree (2) | Not applicable (3) |
| --- | --- | --- | --- |
| I didn't feel like my vote made a difference (2) |  |  |  |
| I was too busy with my personal life (3) |  |  |  |
| I was working and unable to find time (4) |  |  |  |
| I forgot to register to vote (5) |  |  |  |
| I forgot to vote (6) |  |  |  |
| Other (8) |  |  |  |

| Page Break |  |
| --- | --- |

Q27 Did you vote in any governmental election when you were at the following stages of your life?

|  | Yes (1) | No (2) | Not applicable (3) |
| --- | --- | --- | --- |
| College (2) |  |  |  |
| Medical School (3) |  |  |  |
| Residency (4) |  |  |  |

Q28 Which of the following political activities have you previously participated in? Select all that apply.

- Signed a politically oriented petition (1)
- Participated in a tele/ live town hall (4)
- Contacted a politician (5)
- Volunteered for a campaign or political organization (6)
- Donated to a campaign or political organization (7)

End of Block: Voting habits

Start of Block: Early/ Absentee/ Primaries

Q29 Were you aware of these voting options?

|  | I am aware of this (1) | I was not aware of this (5) |
| --- | --- | --- |
| Early voting (1) |  |  |
| Absentee voting (2) |  |  |
| Voting in state primaries or caucus (4) |  |  |

Display This Question:

If Q29 = 1 [ 1 ]

Q30 What has been your past experience with these options?

|  | I have previously done this (2) | I have not previously done this but would consider it in the future (4) | I have not previously done this and am not considering it (7) |
| --- | --- | --- | --- |
| Early voting (1) |  |  |  |

Display This Question:

If Q29 = 2 [ 1 ]

Q31 What has been your past experience with these options?

|  | I have previously done this (2) | I have not previously done this but would consider it in the future (4) | I have not previously done this and am not considering it (7) |
| --- | --- | --- | --- |
| Absentee voting (2) |  |  |  |

Display This Question:

If Q29 = 4 [ 1 ]

Q32 What has been your past experience with these options?

|  | I have previously done this (2) | I have not previously done this but would consider it in the future (4) | I have not previously done this and am not considering it (7) |
| --- | --- | --- | --- |
| Voting in state primaries or caucus (4) |  |  |  |

Display This Question:

If Q29 = 2 [ 5 ]

Q33 Are you interested in learning more about these options?

|  | I plan to learn more about this (4) | I do not plan to use this option (5) |
| --- | --- | --- |
| Absentee voting (2) |  |  |

Display This Question:

If Q29 = 1 [ 5 ]

Q34 Are you interested in learning more about these options?

|  | I plan to learn more about this (4) | I do not plan to use this option (5) |
| --- | --- | --- |
| Early voting (1) |  |  |

Display This Question:

If Q29 = 4 [ 5 ]

Q35 Are you interested in learning more about these options?

|  | I plan to learn more about this (4) | I do not plan to use this option (5) |
| --- | --- | --- |
| Voting in state primaries or caucus (4) |  |  |

| Page Break |  |
| --- | --- |

End of Block: Early/ Absentee/ Primaries

Start of Block: NEMPAC

Q36 Did you know there are emergency medicine political action committees (organizations that  collect money to redistribute to favorable candidates)?

- Yes (1)
- No (6)

Display This Question:

If Q36 = 1

Q37 What is your experience with the emergency medicine political action committees?

- I have contributed (1)
- I'd consider donating if I had more information (4)
- I do not see myself contributing (5)

Display This Question:

If Q36 = 6

Or Q37 = 5

Or Q37 = 4

Q38 Would you like to learn more about the emergency medicine political action committee?

- I'd like to learn more (1)
- I'm not interested in learning more (2)

Skip To: Q40 If Q38 = 2

Display This Question:

If Q38 = 1

Or Q37 = 4

Q39 Please add your email if you would like to learn more about the emergency medicine political action committee:

________________________________________________________________

Display This Question:

If Q37 = 5

Or Q38 = 2

Q40 Please share your thoughts on why you do not see yourself contributing to the emergency medicine political action committee:

________________________________________________________________

________________________________________________________________

________________________________________________________________

________________________________________________________________

________________________________________________________________

End of Block: NEMPAC

Start of Block: Pledge

Q41 Will you make a pledge to vote on or before election day, November 6th 2018?

- Yes (1)
- Maybe (2)
- No (3)

| Page Break |  |
| --- | --- |

Q63 Please provide your email so we can contact you if you win the $200 Amazon gift card drawing.

________________________________________________________________

Q65 What is your name? First Last *(This helps us ensure the survey is not taken multiple times.)*

________________________________________________________________

End of Block: Pledge

# Appendix 3: STROBE Checklist

****Note: page numbers below are the manuscript without the Figures included*****

|  | Item No | Recommendation | Page  No |
| --- | --- | --- | --- |
| **Title and abstract** | 1 | (*a*) Indicate the study’s design with a commonly used term in the title or the abstract | Title |
|  |  | (*b*) Provide in the abstract an informative and balanced summary of what was done and what was found | 1 |
| Introduction | | | |
| Background/rationale | 2 | Explain the scientific background and rationale for the investigation being reported | 3 |
| Objectives | 3 | State specific objectives, including any prespecified hypotheses | 4 |
| Methods | | | |
| Study design | 4 | Present key elements of study design early in the paper | 4 |
| Setting | 5 | Describe the setting, locations, and relevant dates, including periods of recruitment, exposure, follow-up, and data collection | 4 |
| Participants | 6 | (*a*) *Cohort study*—Give the eligibility criteria, and the sources and methods of selection of participants. Describe methods of follow-up  *Case-control study*—Give the eligibility criteria, and the sources and methods of case ascertainment and control selection. Give the rationale for the choice of cases and controls  *Cross-sectional study*—Give the eligibility criteria, and the sources and methods of selection of participants | 4 |
|  |  | (*b*) *Cohort study*—For matched studies, give matching criteria and number of exposed and unexposed  *Case-control study*—For matched studies, give matching criteria and the number of controls per case | NA |
| Variables | 7 | Clearly define all outcomes, exposures, predictors, potential confounders, and effect modifiers. Give diagnostic criteria, if applicable | 5 |
| Data sources/ measurement | 8* | For each variable of interest, give sources of data and details of methods of assessment (measurement). Describe comparability of assessment methods if there is more than one group | *5* |
| Bias | 9 | Describe any efforts to address potential sources of bias | 5 |
| Study size | 10 | Explain how the study size was arrived at | NA |
| Quantitative variables | 11 | Explain how quantitative variables were handled in the analyses. If applicable, describe which groupings were chosen and why | 6 |
| Statistical methods | 12 | (*a*) Describe all statistical methods, including those used to control for confounding | 6 |
|  |  | (*b*) Describe any methods used to examine subgroups and interactions | 7 |
|  |  | (*c*) Explain how missing data were addressed | 7 |
|  |  | (*d*) *Cohort study*—If applicable, explain how loss to follow-up was addressed  *Case-control study*—If applicable, explain how matching of cases and controls was addressed  *Cross-sectional study*—If applicable, describe analytical methods taking account of sampling strategy | NA |
|  |  | (*e*) Describe any sensitivity analyses | NA |

Continued on next page

| Results | | | |
| --- | --- | --- | --- |
| Participants | 13* | (a) Report numbers of individuals at each stage of study—eg numbers potentially eligible, examined for eligibility, confirmed eligible, included in the study, completing follow-up, and analysed | 8 |
|  |  | (b) Give reasons for non-participation at each stage | (13a) |
|  |  | (c) Consider use of a flow diagram | NA |
| Descriptive data | 14* | (a) Give characteristics of study participants (eg demographic, clinical, social) and information on exposures and potential confounders | 8 |
|  |  | (b) Indicate number of participants with missing data for each variable of interest | (figure/ tables/ proportions list denominators) |
|  |  | (c) *Cohort study*—Summarise follow-up time (eg, average and total amount) | NA |
| Outcome data | 15* | *Cohort study*—Report numbers of outcome events or summary measures over time | *NA* |
|  |  | *Case-control study—*Report numbers in each exposure category, or summary measures of exposure | *NA* |
|  |  | *Cross-sectional study—*Report numbers of outcome events or summary measures | (figure/ tables/ proportions numberators) |
| Main results | 16 | (*a*) Give unadjusted estimates and, if applicable, confounder-adjusted estimates and their precision (eg, 95% confidence interval). Make clear which confounders were adjusted for and why they were included | 11-14, 95% CI deferred |
|  |  | (*b*) Report category boundaries when continuous variables were categorized | NA |
|  |  | (*c*) If relevant, consider translating estimates of relative risk into absolute risk for a meaningful time period | NA |
| Other analyses | 17 | Report other analyses done—eg analyses of subgroups and interactions, and sensitivity analyses | 10,11 |
| Discussion | | | |
| Key results | 18 | Summarise key results with reference to study objectives | 14,15,16 |
| Limitations | 19 | Discuss limitations of the study, taking into account sources of potential bias or imprecision. Discuss both direction and magnitude of any potential bias | 16,17 |
| Interpretation | 20 | Give a cautious overall interpretation of results considering objectives, limitations, multiplicity of analyses, results from similar studies, and other relevant evidence | 15,16 |
| Generalisability | 21 | Discuss the generalisability (external validity) of the study results | 17 |
| Other information | | | |
| Funding | 22 | Give the source of funding and the role of the funders for the present study and, if applicable, for the original study on which the present article is based | Title page |
